# Deciduous dental caries status and associated risk factors among preschool children in Xuhui District of Shanghai, China

**DOI:** 10.1186/s12903-018-0565-8

**Published:** 2018-06-19

**Authors:** Hongru Su, Renren Yang, Qinglong Deng, Wenhao Qian, Jinming Yu

**Affiliations:** 1 Xuhui District Dental Centre, Shanghai, China; 2 Collaborative Innovation Centre of Social Risks Governance in Health, School of Public Health, Fudan University, Shanghai, China

**Keywords:** Deciduous dental caries, Preschool children, Risk factors, Logistic models

## Abstract

**Background:**

This study aims to understand the deciduous dental caries status of preschool children in Xuhui District of Shanghai, China and to analyze the associated risk factors.

**Methods:**

In January of 2016, a cross-sectional investigation was conducted to examine the oral health of all the kindergarten children in Xuihui District of Shanghai, China. Meanwhile, a field questionnaire survey was conducted with the children’s guardians to ascertain the potential risk factors associated with deciduous dental caries.

**Results:**

Among 11,153 children, the prevalence of deciduous dental caries was 47.02%, and the mean dmft score was 2.21. The first three predilection sites were maxillary central primary incisors, mandible second primary molars, and mandible first primary molars. There were statistically significant differences in caries prevalence and dmft among different age groups and different household registration (Hukou) types (*P* < 0.001). Multivariate Logistic regression suggested that the possible risk factors for deciduous caries included: older age, drinking sweetened beverages frequently, often or usually eating sweets before sleep compared to rarely/never eat them at this time, exclusive or predominant breastfeeding compared to exclusive or predominant artificial feeding and latter introduction of toothbrushing. On the other hand, Shanghai Hukou families, high educational level of guardians (high school or college education), regular parental support for children’s toothbrushing, guardians’ oral health knowledge, and a good perception about children’s oral health conditions were shown as potential protective factors for deciduous dental caries.

**Conclusions:**

The deciduous dental caries status of preschool children in Xuhui District of Shanghai was still serious. The caries prevalence in Xuhui, China, is associated with children’s age, household registration type, oral health habits, feeding habits, guardians’ education level, parental perception about children’s oral health and knowledge about oral health.

**Electronic supplementary material:**

The online version of this article (10.1186/s12903-018-0565-8) contains supplementary material, which is available to authorized users.

## Background

Early childhood caries (ECC) is defined as the presence of one or more decayed, missing, or filled surfaces in any primary tooth in children younger than 6 years of age [1]. It is a serious public health problem that adversely affects children’s physical and mental health, since dental decay can cause pain, reduced growth and development, speech disorders, and premature tooth loss that lead to chewing problems, loss of self-confidence, and harm to the permanent dentition [2]. The World Health Organization (WHO) lists dental caries as the third most common chronic, non-infectious disease after cancer and cardiovascular disease [3].

In recent years, the material living standard in China has increased a lot; however, oral health services have not improved accordingly, and many people lack oral health knowledge, resulting in a high prevalence of dental caries in China, especially among children. According to the 3rd National Oral Health Survey [4], the caries prevalence among 5-year-old children in 30 provinces of China was 66.0%. The prevalence among urban and rural children was 62 and 70.2%, respectively. The average caries was 3.50. This was an improvement over the results of the 2nd National Oral Health Survey, which was conducted in 1995 (caries prevalence: 76.6%, average caries: 4.5). However, there still exists a large gap between the current status and the WHO’s goal-a 90% caries free population of 5-year-old children by 2010.

When compared to some developed countries, the caries prevalence among Chinese children was also at a very high level. For instance, the American National Health and Nutrition Examination Survey (NHANES 1999–2004) showed that the caries prevalence among 5-year-old children in America was 28% [5]. An oral health survey in England in 2003 revealed that the caries prevalence among British children was 43% [6]. In 2009, a study suggested that the caries prevalence was 37% among 4–5 years old children in Singapore [7]. From these results, we can conclude that greater prevention efforts targeting childhood caries are urgently needed in China, especially among preschool children. Since deciduous teeth are the major mastication organs of preschool children; if these teeth are carious, masticatory function is negatively affected, which not only blocks the intake of nutrients but also harms the growth of permanent teeth and even damages the oral mucosa. Hence, deciduous caries has a negative impact on children’s growth and development.

Xuhui District is in the southwest of central Shanghai, China, which covers a majority of high-income residents. Meanwhile, there are also quite a number of migrant workers from other cities. Even though there have already been several studies regarding dental caries status of the Chinese population, a large sample survey, especially on preschool children in the area like Xuhui District, is still lacking. In this setting, the present study, which covered a large sample of preschool children, aims to understand the epidemiology of deciduous caries through a cross-sectional investigation of kindergarten children in Xuhui District of Shanghai. We hope the findings will be useful for future intervention.

## Methods

### Research design and participants

This present study was carried out together with the “Teeth Fluoridization Program for Children” which covered every kindergartener in Xuhui District and it also served as the baseline investigation of this program. This cross-sectional investigation was conducted in Xuhui District of Shanghai in January 2016. According to a survey in Xuhui District in 2009, the deciduous caries prevalence among 883 kindergarteners was 54.9% [8]. With the admissible error set at 10% of the prevalence, the significance level set at 0.05, and the design effect set at 3.0, the calculated sample size was 947. In view of no response and invalid questionnaires, we expanded the sample size by 10%. As a result, the required minimum sample size turned out to be 1042.

We selected all the community kindergartens in Xuhui District of Shanghai, China via cluster sampling. Every child studying at these kindergartens was included in this study. Those who were younger than 3-year-old or older than 6-year-old were excluded. In addition, the children’s guardians (parents and grandparents) were also included for a questionnaire investigation. The study protocol was approved by the Ethics Committee of Xuhui District Dental Centre.

### Oral health questionnaire

The distribution and collection of these questionnaires was conducted by teachers in each kindergarten who received unified training before the field investigation began. The parents or guardians were asked to complete the questionnaire on the spot the same day before the clinical examination of their children in January 2016. The questionnaire used in this study (refer to the Additional file 1) which was derived from the 4th National Oral Health Survey in China included the following aspects: the education level of guardians, feeding patterns, the daily eating habits and oral health habits of the children, parental perception about child’s oral health, and guardians’ oral health knowledge. Written informed consent was obtained from all guardians who agreed to participate in the study.

### Clinical examination

The dental examinations were conducted in the community kindergartens by trained dentists using a 0.5 mm ball-ended CPI probe and a disposable dental mirror. The results were recorded by another investigator. Dental caries assessments were based on the criteria recommended by the WHO [9], and the dmft index was used to record the caries experiences of the primary dentition. All the examination results were recorded on a caries specified checklist (refer to the Additional file 2) along with the child’s gender, birthdate and household registration. This study involved 14 examiners and all the examiners were experienced dentists who received uniform specialized training before the beginning of the study. Meanwhile, to estimate the intra-examiner agreement on the assessment of caries status, we conducted a pilot survey after the training. The Kappa value was 0.89, which suggested high internal consistency in the dental examination.

### Statistical analysis

Data were entered with Epidata 3.1, analyzed with R 3.4.1, and plotted with GraphPad Prism 6.01. The significance level was set at 0.05. The multiple imputation with MICE [10] in R was conducted for missing data. Observations that contained more than 15% missing values were directly removed from the original data. Statistical description was performed to calculate the prevalence of deciduous caries and the mean dmft score. For enumeration data, the Pearson Chi-square test (nominal data) and the Cochran-Armitage test for trend (ordinal data) were adopted. For continuous data, the following test methods were used, each for a specific situation: t test (two groups), Mann-Whitney test (two groups; and the data did not meet the requirements of parametric test), one-way ANOVA (more than two groups), and Kruskal-Wallis test (more than two groups; and the data did not meet the requirements of parametric test). A binary non-conditional Logistic model was applied to conduct multivariate regression analysis. The dependent variable was dental caries status (1 = carious, 0 = caries free). The independent variables were all the factors surveyed in this study. Some variables were recoded: the education level of guardians was recoded into 3 categories (below high school, high school, college and above), the feeding patterns was recoded into 3 categories (exclusive or predominant artificial feeding, exclusive or predominant breastfeeding, combination); age, the frequency of eating desserts and candies, the frequency of drinking sweetened beverages, the age of starting toothbrushing, parental perception about children’s oral health and score of guardians’ oral health knowledge were included in the model as continuous variables according to questionnaire. Based on the Logistic model, prevalence ratio was estimated using conditional method which was proposed by Wilcosky & Chambless [11].

## Results

### General conditions

Eleven thousand three hundred thirty-five caries checklists and 10,211 questionnaires were obtained during the present investigation. After removing invalid checklists, 11,153 remained; the validity rate was 98.39%. Then, we merged the caries checklists with the questionnaires by ID, removing the invalid ones. There were 9804 remaining after this step.

Among 11,153 children, 5972 (53.55%) were boys and 5181 (46.45%) were girls. The age ranged from 3 to 6 years old and the average was (4.87 ± 0.89) years old. Among these children, 10,700 (95.94%) held a Shanghai Hukou, while 453 (4.06%) did not. In total, 91.82% of the children had biological parents as guardians. As for the guardians, 5.86% reported below high school education levels, 12.00% reported high school education levels and 82.14% reported college and above education levels.

### Deciduous dental caries status

**Fig. 1:**
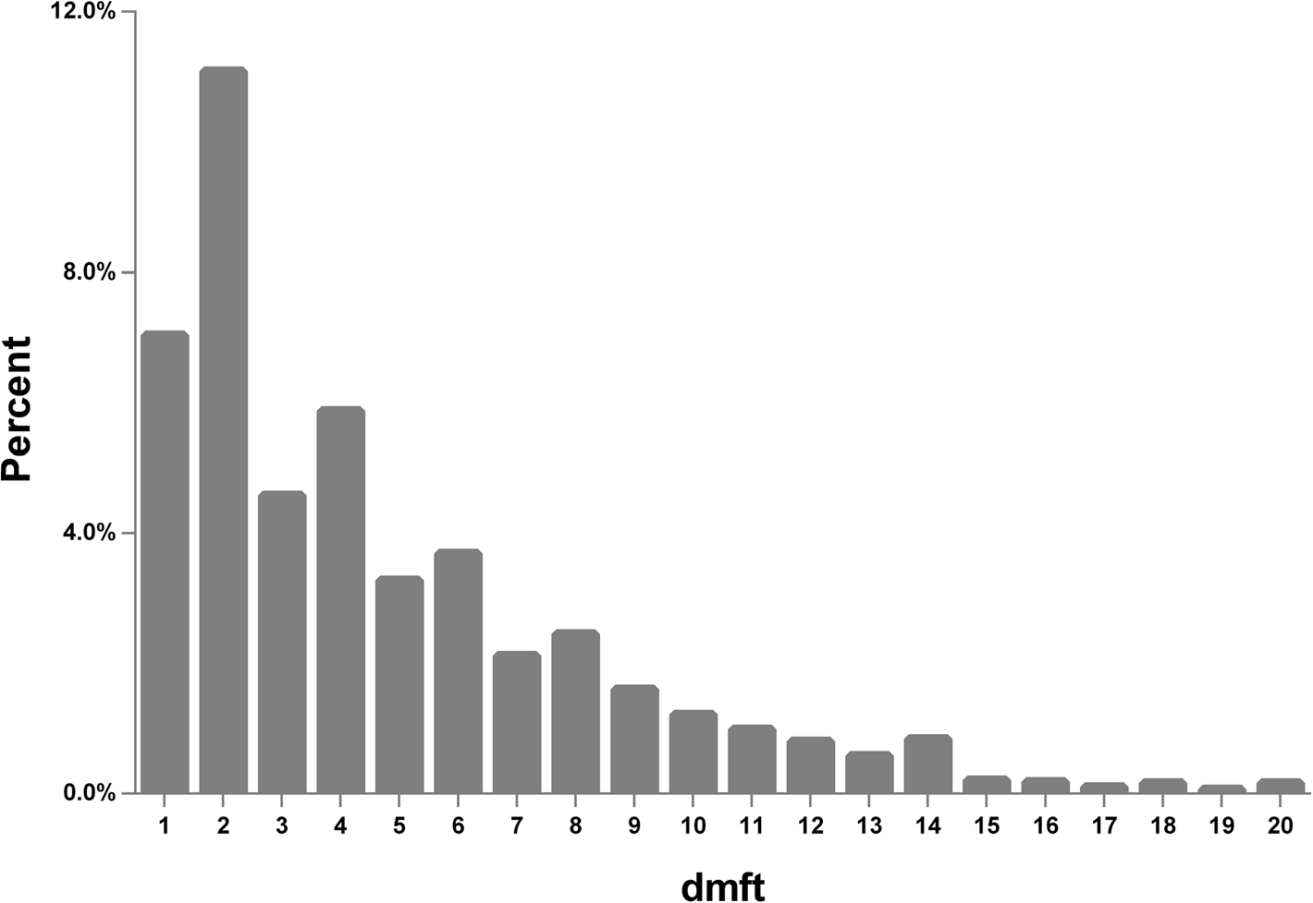
The frequency distribution of dmft

The prevalence of deciduous dental caries was 47.02%. The mean dmft score of the total population was 2.21 and of patients was 4.70. The predilection sites of caries were, in order, maxillary primary central incisors (5684), mandible second primary molars (4566), mandible first primary molars (4383), maxillary second primary molars (2977), maxillary first primary molars (2550), maxillary primary lateral incisors (2342), maxillary primary canines (975), mandible primary canines (470), mandible primary central incisors (324), and mandible primary lateral incisors (271). The deciduous caries status among different age groups, genders and household registration types was shown in Table 1. From the results, we can find that there were significant differences in caries prevalence and dmft between different age groups and different household registration types (*P* < 0.001). However, there were no significant differences in caries prevalence and dmft between genders (*P* = 0.702; *P* = 0.574). The frequency distribution of dmft was as shown in Fig. 1 (the bar for “dmft = 0” was removed). Children with 2 decayed teeth were the most frequent (11.08%), followed by those with 1 decayed tooth (7.04%) and 4 decayed teeth (5.87%).

**Table 1:** Deciduous caries status of different age groups, genders and household registration types (*n* = 11,153)

Variables	Categories	Caries prevalence			dmft	
		No. of surveyed (No. of cases with dental caries)	Caries prevalence (%)	*P*-value (statistics^1^)	Mean ± SD	*P*-value (statistics^2^)
Age (years old)	3	531 (156)	29.38	< 0.001** (−15.992^a^)	1.09 ± 2.45	< 0.001** (304.022^b^)
	4	3626 (1141)	38.91		1.68 ± 3.08	
	5	3798 (1908)	50.24		2.45 ± 3.57	
	6	3198 (1769)	55.31		2.71 ± 3.69	
Gender	Boys	5972 (2818)	47.19	0.702 (0.146)	2.23 ± 3.43	0.574 (0.562^c^)
	Girls	5181 (2426)	46.82		2.19 ± 3.45	
Household registration	Shanghai	10,700 (4959)	46.35	< 0.001** (47.890)	2.14 ± 3.38	< 0.001** (−8.806^d^)
	Non-Shanghai	453 (285)	62.91		3.76 ± 4.43	
Total	–	11,153 (5244)	47.02	–	2.21 ± 3.44	–

**P < 0.01. SD = standard deviation

^1^a: Cochran-Armitage test for trend (the statistics is Z-value); the others were Pearson Chi-square test (the statistics is χ^2^-value)

^2^b: Kruskal-Wallis test (the statistics is χ^2^-value); c: t test (the statistics is t-value); d: Mann-Whitney test (the statistics is Z-value)

Deciduous caries prevalence of the surveyed children categorized by different factors of the questionnaire was as shown in Table 2. Except for guardians (*P* = 0.076) and the frequency of toothbrushing (*P* = 0.063), every other factor revealed a significant difference in caries prevalence.

**Table 2:** Deciduous caries prevalence of the surveyed children categorized by different factors of the questionnaire (*n* = 9804)

Variables	Categories	No. of surveyed (No. of cases with dental caries)	Caries prevalence (%)	*P*-value (statistics^1^)
Guardians	Parents	9002 (4196)	46.61	0.076 (3.149^a^)
	Grandparents	802 (400)	49.88	
Education level of guardians	Illiteracy	26 (19)	73.08	< 0.001** (13.436)
	Primary school	52 (36)	69.23	
	Middle school	497 (310)	62.37	
	High school	704 (403)	57.24	
	Technical secondary school	472 (270)	57.20	
	Junior college	2065 (1033)	50.02	
	Undergraduate	4532 (1958)	43.20	
	Graduate and above	1456 (567)	38.94	
Feeding patterns	Exclusive breastfeeding	3162 (1570)	49.65	< 0.001** (20.015^a^)
	Predominant breastfeeding	2057 (934)	45.41	
	Exclusive artificial feeding	1133 (550)	48.54	
	Predominant artificial feeding	919 (402)	43.74	
	Combination feeding	2533 (1140)	45.01	
Eating desserts and candies (times per week)	< 1	503 (216)	42.94	< 0.001** (−4.432)
	1–2	908 (401)	44.16	
	3–4	1272 (595)	46.78	
	5–6	3217 (1427)	44.36	
	7–8	2824 (1403)	49.68	
	> 8	1080 (554)	51.30	
Drinking sweetened beverages (times per week)	< 1	3544 (1465)	41.34	< 0.001** (−8.810)
	1–2	2548 (1219)	47.84	
	3–4	1806 (890)	49.28	
	5–6	1259 (672)	53.38	
	7–8	506 (265)	52.37	
	> 8	141 (85)	60.28	
Eating sweets before sleep (days per week)	Rarely/never (< 1)	4079 (1563)	38.32	< 0.001** (13.962)
	Sometimes (1–3)	5132 (2696)	52.53	
	Often (> = 4)	593 (337)	56.83	
The age of starting tooth- brushing (years old)	< 1	540 (183)	33.89	< 0.001** (−8.635)
	1	1424 (598)	41.99	
	2	3304 (1503)	45.49	
	3	2915 (1483)	50.87	
	> = 4	1621 (829)	51.14	
Toothbrush (times per day)	< 1	5096 (2414)	47.37	0.063 (1.860)
	1	3562 (1683)	47.25	
	> = 2	1146 (499)	43.54	
Helping children brush teeth (days per week)	Rarely/never (< 1)	1984 (984)	49.60	0.001** (−3.332)
	Sometimes (1–3)	4480 (2115)	47.21	
	Often (4–6)	276 (124)	44.93	
	Everyday (7)	3064 (1373)	44.81	
Parental perception about children’s oral health (score)	Poor (1–2)	800 (701)	87.63	< 0.001** (36.423)
	Fair (3)	2759 (1794)	65.02	
	Good (4–5)	6245 (2101)	33.64	
Score of guardians’ oral health knowledge	0–4	385 (182)	47.27	< 0.001** (3.516)
	5–9	1506 (791)	52.52	
	10–14	7913 (3623)	45.79	
Total	–	9804 (4596)	46.88	–

**P < 0.01

^1^a: Pearson Chi-square test (the statistics is χ^2^-value); the others were Cochran-Armitage test for trend (the statistics is Z-value)

### Univariate analysis of risk factors associated with deciduous caries prevalence

Univariate analysis indicated that the possible risk factors of deciduous caries included: older age, non-Shanghai Hukou, low education level of guardians, unhealthy eating habits, latter introduction of toothbrushing, a bad perception about children’s oral health conditions and low scores of guardians’ oral health knowledge. The cPRs and 95% CIs of these factors were listed in Table 3.

**Table 3:** Univariate analysis of risk factors associated with deciduous caries prevalence (n = 9804)

Variables	Categories	β	Z-value	*P*-value	cPR	cPR 95% CI
Age	–	0.371	−15.710	< 0.001**	1.370	1.305–1.439
Gender	Boys					
	Girls	−0.033	− 0.817	0.414	0.983	0.942–1.025
Household registration	Non-Shanghai					
	Shanghai	−0.716	−6.766	< 0.001**	0.722	0.668–0.780
Guardians	Parents					
	Grandparents	0.131	1.774	0.076	1.070	0.995–1.151
Education level of guardians	Below high school	Ref	Ref	Ref		
	High school	−0.262	−2.497	0.013*	0.865	0.768–0.974
	College and above	−0.787	−8.791	< 0.001**	0.692	0.644–0.743
Feeding patterns	exclusive or predominant artificial feeding	Ref	Ref	Ref		
	exclusive or predominant breastfeeding	0.064	1.218	0.223	1.034	0.980–1.092
	Combination feeding	−0.056	− 0.938	0.348	0.971	0.911–1.033
Frequency of eating desserts and candies	–	0.069	4.427	< 0.001**	1.042	1.021–1.063
Frequency of drinking sweetened beverages	–	0.138	8.778	< 0.001**	1.085	1.064–1.108
Eating sweets before sleep (days per week)	Rarely/never (< 1)	Ref	Ref	Ref		
	Sometimes (1–3)	0.577	13.541	< 0.001**	1.362	1.302–1.426
	Often (> = 4)	0.751	8.443	< 0.001**	1.402	1.314–1.497
The age of starting tooth- brushing	–	0.162	8.607	< 0.001**	1.110	1.079–1.141
Toothbrush (times per day)	< 1	Ref	Ref	Ref		
	1	−0.005	−0.112	0.911	0.997	0.953–1.044
	> = 2	− 0.154	−2.346	0.019	0.919	0.855–0.988
Helping children brush teeth (days per week)	Rarely/never (< 1)	Ref	Ref	Ref		
	Sometimes (1–3)	−0.096	−1.772	0.076	0.950	0.898–1.006
	Often (4–6)	−0.187	− 1.452	0.146	0.901	0.779–1.043
	Everyday (7)	−0.192	−3.328	0.001	0.901	0.847–0.959
Parental perception about children’s oral health	–	−0.957	−33.800	< 0.001**	0.952	0.947–0.958
Score of guardians’ oral health knowledge	–	−0.030	−4.186	< 0.001**	0.987	0.981–0.992

*cPR* crude Prevalence Ratio, *CI* Confidence Interval, *Ref* Reference group

* *P* < 0.05; ** *P* < 0.01

### Multivariate Logistic regression analysis of risk factors associated with deciduous caries prevalence

Higher prevalence of dental caries had been associated with older age, non-Shanghai Hukou, low education level of guardians, rare or inexistent parental support for children toothbrushing, a bad perception of children’s oral health, low scores of guardians’ oral health knowledge and unhealthy eating habits, as frequent ingestion of sweetened beverages or regular consumption of sweets at night or before sleeping. Meanwhile, children who started toothbrushing at earlier age and brushed their teeth more than once daily were with less caries experience. In addition, children who were exclusively or predominantly formula-fed had significantly lower caries prevalence than those exclusively or predominantly breastfed. The aPRs and 95% CIs of these factors were listed in Table 4.

**Table 4:** Association between caries and independent variables in Xuhui, China (*n* = 9804)

Variables	Categories	β	Z-value	*P*-value	aPR	aPR 95% CI
Age	–	0.351	12.875	< 0.001**	1.342	1.269–1.419
Gender	Boys					
	Girls	−0.015	− 0.331	0.741	0.992	0.946–1.040
Household registration	Non-Shanghai					
	Shanghai	−0.347	−2.811	0.005**	0.843	0.756–0.941
Guardians	Parents					
	Grandparents	0.051	0.589	0.556	1.027	0.940–1.122
Education level of guardians	Below high school	Ref	Ref	Ref	Ref	
	High school	−0.219	−1.884	0.060	0.886	0.777–1.010
	College and above	−0.624	−5.900	< 0.001**	0.740	0.677–0.810
Feeding patterns	exclusive or predominant artificial feeding	Ref	Ref	Ref	Ref	
	exclusive or predominant breastfeeding	0.147	2.519	0.012*	1.081	1.017–1.150
	Combination feeding	0.001	0.019	0.985	1.001	0.934–1.072
Frequency of eating desserts and candies	–	0.004	0.200	0.842	1.002	0.982–1.022
Frequency of drinking sweetened beverages	–	0.062	3.289	0.001**	1.035	1.013–1.058
Eating sweets before sleep (days per week)	Rarely/never (< 1)	Ref	Ref	Ref	Ref	
	Sometimes (1–3)	0.367	7.502	< 0.001**	1.216	1.155–1.281
	Often (> = 4)	0.553	5.469	< 0.001**	1.298	1.196–1.409
The age of starting tooth- brushing	–	0.124	5.010	< 0.001**	1.079	1.043–1.117
Toothbrush (times per day)	< 1	Ref	Ref	Ref	Ref	
	1	−0.100	−1.979	0.048*	0.948	0.899–1.000
	> = 2	−0.516	−5.527	< 0.001**	0.741	0.661–0.831
Helping children brush teeth (days per week)	Rarely/never (< 1)	Ref	Ref	Ref	Ref	
	Sometimes (1–3)	−0.188	−2.732	0.007**	0.905	0.841–0.972
	Often (4–6)	−0.134	−0.910	0.362	0.929	0.790–1.093
	Everyday (7)	−0.031	−0.411	0.681	0.983	0.907–1.066
Parental perception about children’s oral health	–	−0.939	−32.053	< 0.001**	0.951	0.945–0.956
Score of guardians’ oral health knowledge	–	−0.017	−2.007	0.045*	0.992	0.985–0.999
Constant	–	2.045	7.140	< 0.001**	–	–

The *P* value of goodness of fit test was 0.062

*aPR* adjusted prevalence ratio, *CI* confidence interval, *Ref* reference group

*P < 0.05; **P < 0.01

## Discussion

This cross-sectional study described deciduous dental caries status quo and tried to assess factors affecting on preschool children’s oral health in Xuhui District. In spite of recent improvement in awareness of oral health among public, dental caries remains a significant problem especially in developing countries. In this study, we discovered that 47.02% of involved preschool children had caries experience. This finding was similar to that of other developing countries such as India (53%) [12] and South Africa (49%) [13]. Caries prevalence found in this investigation was lower than those of an oral health survey conducted in Xuhui District in 2009 (54.9%) [8] as well as a survey among 5-year-olds in Shanghai (66.42%) [14]. Furthermore, this prevalence was below the national average and the average of Shanghai.

Although we can conclude that there was a decrease in caries prevalence among preschool children in Xuhui District from these comparisons, it still had a very high prevalence of dental caries compared with the developed countries mentioned above, which suggested that deciduous dental caries was a serious oral health threat for preschool children. Since dental caries may not be eliminated but can be prevented, the result will be fruitful when appropriate prevention programs are implemented. Accordingly, a coordinated effort among health care providers, policy makers, and health institutions is desperately in need to minimize the prevalence of the disease.

It has been identified previously that socioeconomic status, education level of guardians and oral hygiene and eating habits of children were associated with dental caries [15–17]. This was in agreement with the current study. In our study, a strong association between age and dental caries was found. It was observed the caries severity gradually increased with age in primary dentition, which was consistent with many previous studies [17–19]. This is because caries is a cumulative process. This indicates that health promotion for the caries of dental prevention should begin in the first year of life among children in case the decayed teeth become too advanced to prevent.

Although American Academy of Pediatric Dentistry recommended that toothbrushing should be performed for children twice daily and started as soon as the first primary tooth erupted [1], only 48.02% of those in our study had brushed their teeth once a day or more and most of them had started tooth brushing at 2 years old or older. The children’s dental behaviors have a significant influence on their oral health related quality of life and are important predictors of dental caries. Therefore, on basis of our findings, parents or guardians should be aware of consequences that not toothbrushing could result in children’s oral health and should help children with toothbrushing since early years.

Our study showed that children who held a Shanghai Hukou had a lower caries prevalence and mean dmft score compared to those who did not (46.35%, 2.14 vs 62.91%, 3.76; *P* < 0.001). This significant difference can be explained by the fact that the parents of children without Shanghai Hukou are mainly migrant workers who come from other relatively underdeveloped cities. Migrant children had less access to services about health and education compared to children with local urban Hukou in Shanghai. For example, these children could only temporarily enrol in public schools as transient students and were not entitled to oral-related public health services funded by the tax revenue such as dental caries filling [20]. These findings were in accordance with a number of earlier studies [20–22] which reported the migrant children had higher prevalence of caries and dmft scores.

In recent years, the number of migrant workers in Shanghai has increased year by year [23]. Generally speaking, the socioeconomic status of this population is low. Having settled down in cities, the migrant children might have more delicious foods, more choice for sweet snacks than before they lived in rural areas, so they had higher risk of dental caries [24, 25]. In addition, according to Liu Chengjun et al.’s [20] survey, some bad oral health-related habits of migrant children such as most of children brushing their teeth less than twice daily and had sweet snacks before sleep without toothbrushing could also contribute to higher prevalence of decayed teeth. Thus, it is necessary for local communities to take targeted prevention measures to promote oral health of migrant population in order to improve the child’s caries status.

This study also attempted to assess the influence of feeding habits on dental caries. It was concluded that there was a significant difference in caries prevalence between different feeding patterns (*P* < 0.001) and the prevalence was highest in the group of exclusive breastfeeding. This was the most controversial finding of this study. As for feeding patterns, a recent longitudinal survey [26] in Japan found that infants who had been breastfed for 6–7 months, both exclusively and partially, were at higher risk of dental caries at 30 months than those who had been exclusively formula-fed. Moreover, a cross-sectional investigation [27] about association between early life factors and dental caries in 5-year-old children involving 31 provinces of Mainland China had similar results. They found that exclusively and predominantly formula-fed children had lower caries experience than exclusively breastfed children. However, several reviews [28–30], contrary to our studies, showed strong evidence that breastfeeding was beneficial for the prevention of dental caries.

The results obtained in the present study may be caused by the fact that the involved children had a prolonged breastfeeding. A study [31] in Brazil by Peres, K.G. et al. revealed that prolonged breastfeeding (when children were breastfed for≥24 months) increased the risk of having dental caries. According to this study [31], the mechanism underlying this process may link to following risk factors such as frequent nocturnal breastfeeding, genes and environmental components modifying the susceptibility to caries in children, cariogenic potential component of human milk. Apart from that, like in most low- and middle-income countries, artificial feeding in China is mostly available to richer families in view of the cost of infant formula, and as such, it may represent a very strong indicator of family socioeconomic position. Adjusting for indicators of household income which were not surveyed in the present study would shed some light on this association and more studies should be conducted in the future. Due to these limitations, further study about association between breastfeeding and deciduous dental caries should be carried out. Meanwhile, other preventive measures should be implemented.

Dental caries is a preventable disease, there are many measures that could be taken to reduce the prevalence of deciduous caries. Oral health knowledge should be disseminated to every family, especially those of migrant workers or those with low education levels. Families and nursery institutions should help children develop good oral hygiene and eating habits. Early intervention programs for preschool children’s oral health behavior should be developed based on the risk factors identified in this study. Most importantly, policy makers should work hand in hand to improve the quality and accessibility of oral health services.

Several limitations could be found in this present study. Firstly, the surveyed population was comprised of the kindergarten students in Xuhui District of Shanghai; therefore, those who did not attend kindergartens (although this population was very small) were not included. Secondly, some potential factors that contributed to dental caries, such as dentist visit [32] and the use of fluoride toothpastes, were not included in this study. We did not survey the use of fluoride toothpaste because many parents expressed uncertainty about toothpaste types when we conducted the pilot investigation. Thirdly, there was an unavoidable self-reporting bias since the children’s guardians completed the self-administered questionnaires. Finally, the present study was a cross-sectional investigation, so causal inference was limited.

## Conclusions

This study found that deciduous caries among preschool children in Xuhui District of Shanghai was still a severe problem. Enhancement of preventive measure at early age should be emphasized by guardian and dental health professionals. Furthermore, the caries prevalence in Xuhui, China, is associated with children’s age, household registration type, oral health habits, feeding habits, guardians’ education level, parental perception about children’s oral health and knowledge about oral health.

## Additional files

Additional file 1: Questionnaire. (PDF 399 kb)

Additional file 2: Caries cheklist. (PDF 138 kb)

## Abbreviations

aPR: Adjusted prevalence ratio
CI: Confidence interval
CPP-ACP: Casein phosphopeptide-amorphous calcium phosphate
cPR: Crude prevalence ratio
SD: Standard deviation
WHO: World Health Organization
