# Supplementary material for: Deciduous dental caries status and associated risk factors among preschool children in Xuhui District of Shanghai, China
Source: BMC Oral Health. 2018 Jun 19;18:111. doi: 10.1186/s12903-018-0565-8 (PMC6009057; doi:10.1186/s12903-018-0565-8)
Supplement: Supplementary file 1 — Questionnaire. (PDF 399 kb) [file 12903_2018_565_MOESM1_ESM.pdf]

the 4th National Oral Health Survey （guardians）

Children ID ☐☐☐☐☐☐☐　　　**Investigator ID** ☐

Date of survey ☐☐☐☐year ☐☐month ☐☐day

Note:

1. Only children’s parents or grandparents can fill out this questionnaire.

2. Please draw “ √ ” in “☐” in front of the corresponding options.

1. You are the child’s ____ ?

   1) ☐Father　2) ☐Mother　3) ☐Grandfather　4) ☐Grandmother

2. What’s your highest education level?

   1）☐Didn’t attend school　2）☐Primary school　3）☐Middle school

   4）☐High school　5）☐Technical secondary school

   6）☐Junior college　7）☐Undergraduate　8）☐Graduate and above

3. What’s the feeding pattern of your child in the first 6 months after birth?

   1）☐Exclusive breastfeeding　2）☐Predominant breastfeeding

   3）☐Exclusive artificial feeding　4）☐Predominant artificial feeding

   5）☐Combination feeding

4. What’s the frequency (times per week) of eating the following food of your child?

	<1	1-2	3-4	5-6	7-8	>8
1）Desserts and candies	☐	☐	☐	☐	☐	☐
2）Sweet beverages	☐	☐	☐	☐	☐	☐

5. Does your child often eat sweets before sleep at night?

   1）☐Often（≥4 days per week）　2）☐Sometimes（1-3 days per week ）

3) ☐ Rarely/never (< one day per week)

6. What's the age of starting tooth brushing of your child?

1) ☐ <1-year-old   2) ☐ 1-year-old   3) ☐ 2-year-old

4) ☐ 3-year-old   5) ☐ ≥4-year-old

7. What's the frequency (times per day) of brushing teeth of your child?

1) ☐ <1   2) ☐ 1   3) ☐ ≥2

8. Do you often help your child brush teeth?

1) ☐ Rarely/never (< one day per week)   2) ☐ Sometimes (1-3 days per week)

3) ☐ Often (4-6 days per week)   4) ☐ Everyday (7 days per week)

9. What's your assessment of your child's oral health condition?

Assessment: Very bad--------Fair-------Very good

Score: 1-------2-------3-------4-------5

☐ ☐ ☐ ☐ ☐

10. Do you think the following statements are true or false?

	True	False	Not sure
1) Oral health is important to everyone's life.	☐	☐	☐
2) It's necessary to do oral health examination regularly.	☐	☐	☐
3) The state of our teeth is inborn, which means it is irrelevant to our protection.	☐	☐	☐
4) The prevention of odontopathy depends on ourselves in the first place.	☐	☐	☐
5) It's very important to protect children's six-year-teeth.	☐	☐	☐
6) The state of children's teeth can be affected by			

mothers' teeth. ☐ ☐ ☐

7) Gingival bleeding is normal when brushing teeth. ☐ ☐ ☐

8) Bacteria can cause gingival inflammation. ☐ ☐ ☐

9) Brushing teeth is useless for the prevention of gingival bleeding. ☐ ☐ ☐

10) Bacteria can cause dental caries. ☐ ☐ ☐

11) Eating sugars can cause dental caries. ☐ ☐ ☐

12) There's no need to treat decayed primary teeth. ☐ ☐ ☐

13) Pit and fissure sealing can prevent children's caries. ☐ ☐ ☐

14) Fluoride is useless for protecting teeth. ☐ ☐ ☐

It's the end. Thanks for your participation!
