# Supplementary material for: Deciduous dental caries status and associated risk factors among preschool children in Xuhui District of Shanghai, China
Source: BMC Oral Health. 2018 Jun 19;18:111. doi: 10.1186/s12903-018-0565-8 (PMC6009057; doi:10.1186/s12903-018-0565-8)
Supplement: Supplementary file 2 — Caries cheklist. (PDF 138 kb) [file 12903_2018_565_MOESM2_ESM.pdf]

the 4th National Oral Health Survey（3-6 years old）

Follow-up No. ☐ **Children ID** ☐☐☐☐☐☐☐ **Investigator ID** ☐

Gender ☐ (1=boys 2=girls) **Household registration** ☐ (1=Shanghai 2=Non-Shanghai)

Date of birth ☐☐☐☐ year ☐☐ month ☐☐ day

Date of survey ☐☐☐☐ year ☐☐ month ☐☐ day

Teeth fluoridization ☐ (1=yes 2=no)

		55	54	53	52	51	61	62	63	64	65	
	16	15	14	13	12	11	21	22	23	24	25	26
Dental crown	☐	☐	☐	☐	☐	☐	☐	☐	☐	☐	☐	☐
Dental crown	☐	☐	☐	☐	☐	☐	☐	☐	☐	☐	☐	☐
	46	45	44	43	42	41	31	32	33	34	35	36
		85	84	83	82	81	71	72	73	74	75	

**Primary teeth**	**Permanent teeth**	**Caries status**
A	0	Sound
B	1	Coronal caries
C	2	Filled w/caries
D	3	Filled, no caries
E	4	Missing due to caries
X	5	Missing for any another reason
F	6	Fissure sealant
G	7	Fixed dental prosthesis/crown, abutment, veneer
X	8	Unerupted
T	T	Trauma
N	9	Not recorded

Immediate (urgent) treatment needed ☐ (1=yes 2=no)

Type of table ☐ (1=original table 2=recheck table)
